# Prediction of independence in bowel function after spinal cord injury: validation of a logistic regression model

**DOI:** 10.1038/s41393-020-00551-y

**Published:** 2020-09-22

**Authors:** Omar Khan, Jetan H. Badhiwala, Michael G. Fehlings

**Affiliations:** 1 Division of Neurosurgery, Department of Surgery, University of Toronto, Toronto, ON Canada; 2 Division of Neurosurgery, Toronto Western Hospital, University Health Network, Toronto, ON Canada

**Keywords:** Outcomes research, Prognosis

## Abstract

**Study design:**

Retrospective analysis of prospectively collected data.

**Objectives:**

Recently, logistic regression models were developed to predict independence in bowel function 1 year after spinal cord injury (SCI) on a multicenter European SCI (EMSCI) dataset. Here, we evaluated the external validity of these models against a prospectively accrued North American SCI dataset.

**Setting:**

Twenty-five SCI centers in the United States and Canada.

**Methods:**

Two logistic regression models developed by the EMSCI group were applied to data for 277 patients derived from three prospective multicenter SCI studies based in North America. External validation was evaluated for both models by assessing their discrimination, calibration, and clinical utility. Discrimination and calibration were assessed using ROC curves and calibration curves, respectively, while clinical utility was assessed using decision curve analysis.

**Results:**

The simplified logistic regression model, which used baseline total motor score as the predictor, demonstrated the best performance, with an area under the ROC curve of 0.869 (95% confidence interval: 0.826–0.911), a sensitivity of 75.5%, and a specificity of 88.5%. Moreover, the model was well calibrated across the full range of observed probabilities and displayed superior clinical benefit on the decision curve.

**Conclusions:**

A logistic regression model using baseline total motor score as a predictor of independent bowel function 1 year after SCI was successfully validated against an external dataset. These findings provide evidence supporting the use of this model to enhance the care for individuals with SCI.

## Introduction

Traumatic Spinal Cord Injury (SCI) is a debilitating condition carrying devastating consequences for patients, families, and society at-large [1, 2]. Individuals with SCI frequently experience neurogenic bowel dysfunction, which may be characterized by stool retention, constipation, and fecal incontinence [3]. In addition to adversely affecting patient quality-of-life [4, 5] and being an enormous burden on the healthcare system [6], neurogenic bowel dysfunction can lead to a host of life-threatening sequelae such as intestinal obstruction, recurrent urinary tract infections from chronic constipation, and hemorrhoidal disease [7, 8].

To limit the harmful effects of these potentially disastrous consequences, clinicians have developed several management modalities for SCI patients with bowel dysfunction, ranging from conservative (targeting diet and bowel habits) to invasive (e.g. sacral anterior root stimulation and permanent colostomy) [8, 9]. Despite these strategies, there is no definitive cure for neurogenic bowel dysfunction, making it a chronic, life-altering condition.

The wide-ranging harms and lasting effects of neurogenic bowel dysfunction make the early prediction of independence in bowel function an important endeavor. With accurate early prediction, clinicians can provide patients with better counseling, better preparation, and potentially better outcomes through earlier intervention. Recently, Pavese et al. [10] developed a full and simplified logistic regression model to predict the probability of independence in bowel function 1 year after SCI. The authors used a 1250-patient European Multicenter SCI (EMSCI) dataset for model development, and a 186-patient EMSCI dataset (consisting of patients enrolled at a later date) for model validation.

While the results of the regression models on both the derivation and validation data were encouraging, validation against an external dataset is a necessary step to fully adopt the model for clinical practice [11]. In this article, we test the performance of the regression models developed by Pavese et al. [10] on an external dataset built from collating SCI data of patients treated at North American centers. Through successful external validation, we hope to add to the foundation built by Pavese et al. that would lead to the application of the models to enhance the care of individuals with SCI.

## Methods

### Study design

This is a retrospective analysis utilizing data derived from combining three prospectively collected datasets on traumatic SCI: the North American Clinical Trials Network (NACTN) SCI registry [12], the Surgical Timing in Acute Spinal Cord Injury Study (STASCIS) [13], and the National Acute Spinal Cord Injury Study (NASCIS III) [14]. Patients were recruited from 2005 to 2017 in the NACTN SCI registry, 2002 to 2009 in the STASCIS trial, and from 1991 to 1995 in the NASCIS III trial. These studies prospectively followed patients with SCI and collected patient characteristics, functional outcome data, and neurological examination data at baseline, as well as functional outcome data 1 year after SCI. Further details regarding patient enrollment, inclusion and exclusion criteria, and interventions employed in the individual datasets can be found in the respective publications [12–14].

### Patient population

From the NACTN, STASCIS, and NASCIS III datasets, we included patients with functional data at baseline and 1-year post-injury, as assessed by the Spinal Cord Independence Measure (SCIM) [15]. SCIM is a validated tool used to assess the degree of independence in various functional domains (e.g. ambulation, bladder function, bowel function) after SCI [15].

In addition, we excluded patients with an incomplete neurological examination at baseline, which was performed according to the International Standards for Neurological Classification of Spinal Cord Injury (ISNCSCI) [16]. In the ISNCSCI system, sensory function in both light touch and pinprick domains is rated for each dermatome in the body on a scale from 0–2, with 0 indicating absence of sensation, 1 indicating altered sensation, and 2 indicating intact sensation. Motor function is evaluated in 5 muscle groups of each limb using a score from 0–5, with 0 representing no motor function and 5 representing completely intact muscle strength against full resistance. With this scale, the maximum upper extremity motor score bilaterally is 50 points, while the maximum lower extremity motor score bilaterally is 50 points, leading to a total possible score of 100. Voluntary anal contraction and sensation of deep anal pressure are also assessed in the ISNCSCI exam. After excluding patients with missing data, 277 patients were used for external validation.

### Outcome measures

The primary outcome measure of this study was independence in bowel function as defined by regular bowel movements requiring no assistance and fewer than 2 episodes per month of bowel incontinence. As the SCIM outcomes recorded in our study were from version II, independence in bowel function was characterized by an item 7 SCIM score of 10. For patients in our study, this outcome was dichotomized in a manner consistent with the study reported by Pavese et al. for SCIM version II [10]. Individuals with an item 7 score of 10 at 1-year post-SCI were assigned an outcome of ‘1’, while those with an item 7 score less than 10 were assigned an outcome of ‘0’.

### Statistical analysis

We applied both the full and simplified logistic regression models developed by Pavese et al. [10] to our 277-patient dataset to predict the probability of independence in bowel function (denoted by *P*) 1 year after SCI. The equations used to calculate *P* from the full regression model are as follows:

1 $$P = \frac{{e^f}}{{1 + e^f}},\,{\mathrm{where}}\,\,f = \beta _1 + \beta _2 \ast M_{tot} + \beta _3 \ast SCIM3a$$

where *β*_1_ = −2.25046, *β*_2_ = 0.0486938, and *β*_3_ = 0.4178468 are constants. Note that SCIM3a represents independence in upper body dressing at baseline (SCIM subscore 3a) while *M*_*tot*_ represents the baseline total motor score, calculated by summing the motor scores for the upper extremity muscle groups and the lower extremity muscle groups. In addition to evaluating the full model, we analyzed the simplified model used by Pavese et al. [10]. In the simplified model, the SCIM3a term was removed; otherwise, it was the same as the full model. The simplified model is represented via the following equation:

2 $$P = \frac{{e^f}}{{1 + e^f}},\,{\mathrm{where}} \,\,f = \beta _1 + \beta _2 \ast M_{tot}$$

Supplementary Table 1 depicts the relationship between the total baseline motor score and the probability predicted by the simplified model. After calculating *P* for every patient in our dataset, we evaluated the validity of both the full and simplified models on our data by comparing *P* to the actual outcome of each patient. With this comparison, we determined the model’s discrimination, calibration, and potential clinical utility. Discrimination refers to the ability of the model to properly distinguish patients who achieved bowel independence from those who did not [17]. Discrimination for both models was assessed using the area under the receiver operating characteristic curve (aROC) and its 95% confidence interval, accuracy, sensitivity, and specificity. An aROC of 1 denotes perfect discrimination, while an aROC of 0.5 denotes no discrimination.

Calibration refers to the consistency between the probabilities predicted by the model (*P*) and the actual probability of 1-year bowel independence observed in the dataset [17, 18]. It is determined using calibration curves and graphical depictions of the relationship between predicted and actual probabilities. Numerically, the slope and intercept of the calibration curve provide information on the degree of calibration, such that a model whose calibration curve has a slope of 1 and an intercept of 0 is considered perfectly calibrated. For both full and simplified models, calibration on our data was evaluated using calibration curves, slopes, and intercepts, along with their 95% confidence intervals.

Finally, we undertook decision curve analysis to determine the potential clinical utility of both full and simplified models [19, 20]. Decision curves are plots of the net clinical benefit of using the model to predict outcomes for various probability thresholds. Net benefit depends on the true positive rate, false-positive rate, prevalence of patients who achieved independent bowel function, and the relative weight assigned to true positive rate over the false-positive rate based on the threshold probability. In a clinical context, the probability threshold may be set by the physician, and is used to determine the clinical utility of a predictive model via decision curve analysis.

To better explain decision curves, we describe an example of a physician with a probability threshold of 60%. This means that a patient whose probability of achieving bowel independence at 1 year is below 60% will be assumed to lack independence in bowel function at 1 year. In contrast, a patient whose probability is above 60% will be assumed to be independent at 1 year. If the physician with a probability threshold of 60% then sees a new patient with spinal cord injury, the physician has two main choices. He/she can initiate default management (used for patients likely to have neurogenic bowel dysfunction) or the physician can use an alternative management regime (typically used for patients likely to exhibit independence in bowel function).

This management decision could be independent of the patient’s individual probability of recovery or could be based on what the physician anticipates will be the probability of recovery (e.g. through using a predictive model). The decision curve is then used to answer the question: “for a given probability threshold, what is the net clinical benefit of using the full or simplified logistic regression model to predict independence in bowel function over a) using default management for every patient and b) using alternative management for every patient?”. Statistical analysis was performed using R version 4.0.2 (The R Foundation, Vienna, Austria) and RStudio version 1.2.1335 (RStudio, Boston, Massachusetts).

## Results

### Patient data

**Fig. 1:**
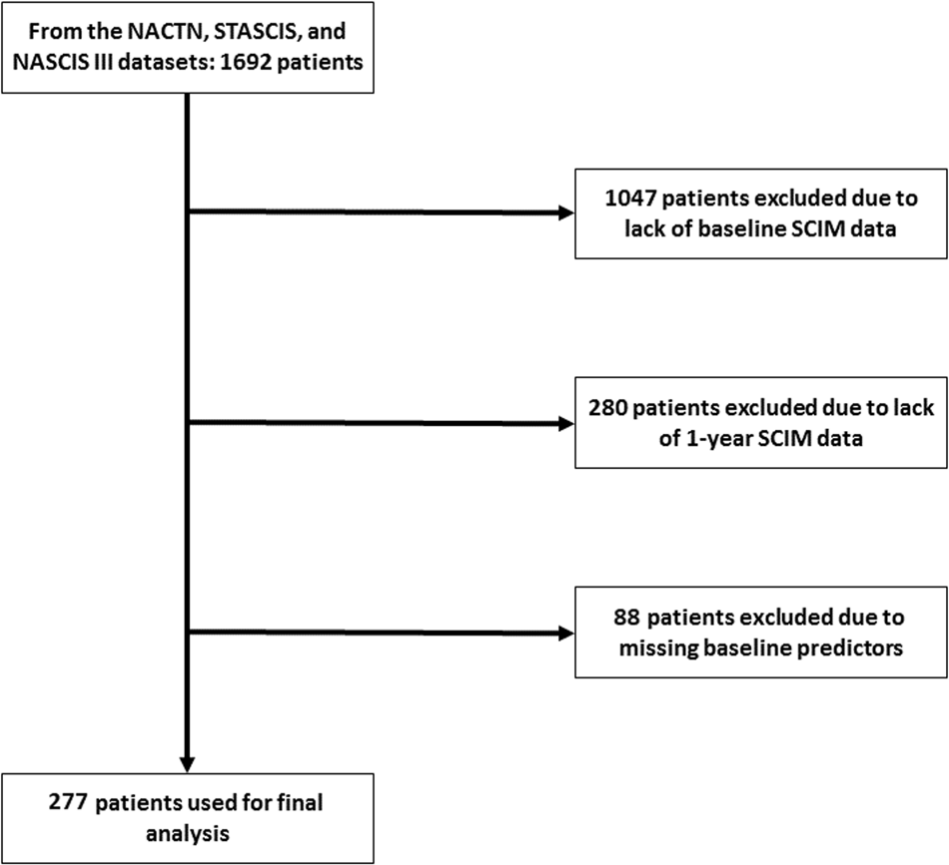
Flow diagram indicating inclusion and exclusion of patient data for final analysis. NACTN North American Clinical Trials Network, STASCIS Surgical Timing in Acute Spinal Cord Injury Study, NASCIS National Acute Spinal Cord Injury Study, SCIM Spinal Cord Independence Measure.

Figure 1 depicts the flow of patients. In total, 1692 patients from the NACTN SCI registry, STASCIS, and NASCIS III trials were available for analysis. However, only 645 patients had SCIM data present at baseline. Of these, 365 patients had bowel outcome data available at the 1-year follow-up. Because of missing predictors (i.e. baseline SCIM3a and total motor score), 88 patients were excluded, leaving 277 patients with complete data for further analysis.

The baseline characteristics of the 277-patient sample are shown in Table 1. The mean age was 46.3 years, and males comprised most of the cohort. The majority of patients had a neurological level of injury at the level of the cervical spine, with an even larger majority being treated surgically for SCI. ASIA Impairment Scale (AIS) D, denoting motor incomplete injury [16], was the most common class of neurological deficit. Slightly over half of patients demonstrated complete independence in bowel function at the 1-year follow-up.

**Table 1:** Baseline clinical characteristics and outcomes of the 277-patient cohort.

Variable	Mean (SD), frequency (percentage), or median (IQR)	Range (minimum–maximum)
Age (Years)	46.3 (16.8)	15–86
Male sex	212 (80.0%)	N/A
Presence of comorbidities	168 (61.1%)	N/A
Hypertension	40 (14.6%)	N/A
Diabetes mellitus	26 (9.5%)	N/A
History of myocardial infarction	4 (1.5%)	N/A
Respiratory comorbidities	12 (4.4%)	N/A
Cancer	1 (0.4%)	N/A
Cerebrovascular disease	3 (1.1%)	N/A
Other comorbidities	108 (41.5%)	N/A
Neurological level of injury		
C1-C8	198 (71.5%)	N/A
T1-T12	46 (16.6%)	N/A
L1-L5	12 (4.3%)	N/A
S1-S5	21 (7.6%)	N/A
Severity of neurological deficit		
AIS A	80 (29.7%)	N/A
AIS B	34 (12.6%)	N/A
AIS C	34 (12.6%)	N/A
AIS D	110 (40.9%)	N/A
AIS E	11 (4.1%)	N/A
Current smoker	68 (24.8%)	N/A
Treated surgically	255 (93.8%)	N/A
Baseline total motor score	52.9 (32.7)	0–100
Baseline SCIM3a score (upper body dressing)	1 (2)	0–3
Baseline SCIM respiration and sphincter management	22.5 (12.9)	0–40
Baseline total SCIM score	43.3 (31.3)	0–99
Days between injury and baseline evaluation	3.9 (17.1)	0–197
Independence in bowel function at 1 year	155 (56.0%)	N/A

Continuous variables are represented using mean (standard deviation or SD), categorical variables are represented using frequency and percentage (%), and ordinal variables (e.g. SCIM3a) are represented using median and interquartile range.

*SCIM* Spinal Cord Independence Measure, *AIS* American spinal injury association Impairment Scale, *SD* standard deviation, *IQR* Interquartile Range, *N/A* not applicable.

### Validation of full model

**Fig. 2:**
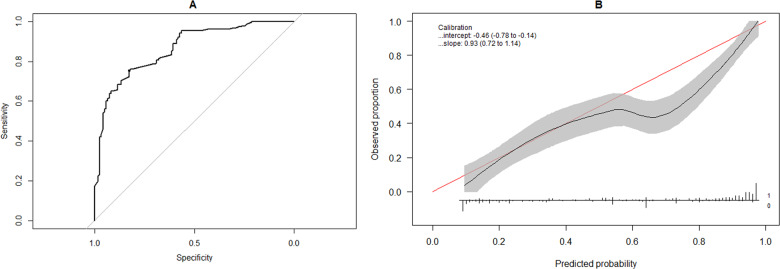
Discrimination and calibration of full logistic regression model (Equation 1). **a** Receiver operating characteristic (ROC) curve of the full logistic regression model applied to the external validation dataset. Gray line denotes a model with an area under the ROC curve of 0.5 (i.e. zero discriminative ability). **b** Calibration curve (black line) comparing observed probabilities in the external validation dataset to probabilities predicted by the full logistic regression model. Shaded gray area denotes the pointwise 95% confidence limits of the calibration curve. Horizontal line denotes the range of probabilities predicted by the model, while vertical bars on this line denote the relative numbers of patients who exhibited independence in bowel function at 1 year (‘1’) or did not exhibit independence in bowel function (‘0’) for each predicted probability.

The full model (Eq. (1)) was first applied to our dataset, and its receiver operating characteristic curve (Fig. 2a) and calibration curve (Fig. 2b) were determined. The full model demonstrated an aROC of 0.864 (95% confidence interval: [0.822,0.906]) with an accuracy of 78.7%, a sensitivity of 75.5%, a specificity of 82.8%, a positive predictive value of 84.8%, and a negative predictive value of 72.7%. The calibration curve of the full model had a slope of 0.93 and an intercept of −0.46. From Fig. 2b, the model displayed acceptable calibration at lower observed probabilities. However, at larger observed probabilities, the full model tended to overestimate the chances of complete independence in bowel function.

**Fig. 3:**
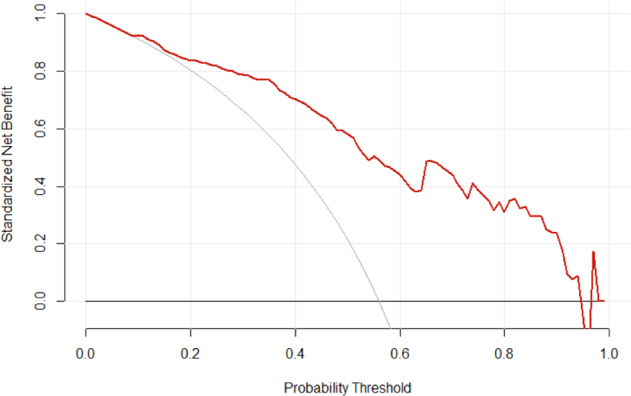
Decision curve analysis of the full regression model. The graph shows the net benefit at different probability thresholds of using the full regression model (red curve) relative to managing all spinal cord injury patients in the default manner (horizontal line) or alternative manner (gray curve).

Figure 3 shows the decision curve of the full model with respect to our data. At lower probability thresholds, the net clinical benefit of using the regression model to predict probability of recovery is roughly equivalent to employing alternative management for every patient (meant for the patients likely to recover). However, at larger probability thresholds, the net benefit of using the regression model exceeds the net benefit of employing alternative management or employing default management for every patient (with the exception of a minor dip at a threshold probability of around 0.95). Taken together, these results suggest that the full model displays adequate performance on our data, except for the miscalibration at larger observed probabilities.

### Validation of simplified model

**Fig. 4:**
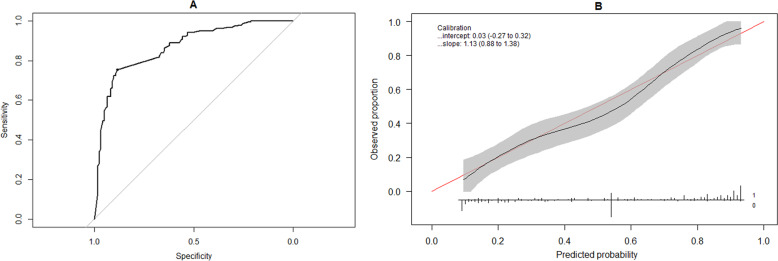
Discrimination and calibration of the simplified logistic regression model (Equation 2). **a** Receiver operating characteristic (ROC) curve of the simplified logistic regression model applied to the external validation dataset. Gray line denotes a model with an area under the ROC curve of 0.5 (i.e. zero discriminative ability). **b** Calibration curve (black line) comparing observed probabilities in the external validation dataset to probabilities predicted by the simplified logistic regression model. Shaded gray area denotes the pointwise 95% confidence limits of the calibration curve. Horizontal line denotes the range of probabilities predicted by the model, while vertical bars on this line denote the relative numbers of patients who exhibited independence in bowel function at 1 year (‘1’) or did not exhibit independence in bowel function (‘0’) for each predicted probability.

**Fig. 5:**
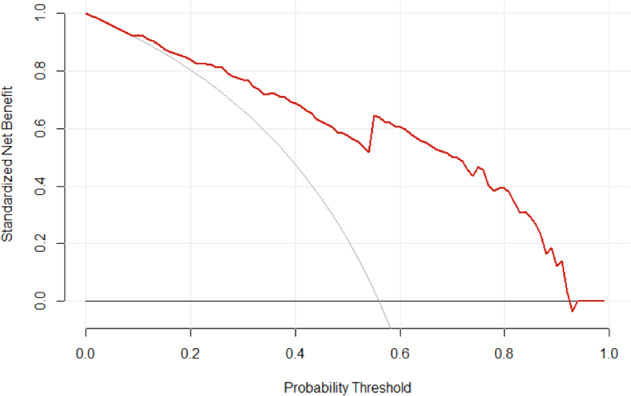
Decision curve analysis of the simplified regression model. The graph shows the net benefit at different probability thresholds of using the simplified regression model (red curve) relative to managing all spinal cord injury patients in the default manner (horizontal line) or alternative manner (gray curve).

Figure 4a, b show the ROC curve and calibration of the simplified model on our dataset, respectively. The simplified model demonstrated an aROC of 0.869 (95% confidence interval: [0.826,0.911]) with an accuracy of 81.2%, a sensitivity of 75.5%, a specificity of 88.5%, a positive predictive value of 89.3%, and a negative predictive value of 74.0%. The calibration curve demonstrated a slope of 1.13 with an intercept of 0.03, and unlike the calibration plot for the full model, displayed good calibration across the full range of observed probabilities. Finally, decision curve analysis (Fig. 5) of the simplified model gave results similar to those of the full model, with the net clinical benefit of the regression model exceeding that of alternative options.

## Discussion

This study evaluated the external validity of two regression models proposed by Pavese et al. [10] to predict the likelihood of achieving independence in bowel function 1 year after SCI. The simplified regression model using only the total baseline motor score as a predictor had good discrimination, was well calibrated, and showed promising clinical utility when applied to our dataset. Meanwhile, the full regression model, which used both baseline total motor score and baseline upper body dressing ability as predictors, showed good discrimination and clinical utility; however, it was not perfectly calibrated at larger observed probabilities.

In the original article that developed these models [10], both the full and simplified models displayed good discrimination and good calibration on the derivation cohort. However, the full model had marginally better performance against the derivation cohort used by the authors (aROC of 0.848 vs 0.837 for the simplified model). Despite this small difference in performance, our external validation study demonstrated that the simplified model was superior to the full model due to its better calibration.

To the authors’ knowledge, a limited number of articles in the literature have successfully developed predictive models for independence in the functional domains affected by traumatic SCI. These articles have primarily focused on independence in ambulation [21], upper limb function [22], bladder function [23], and bowel function [10]. Further, only the studies that have created prognostic models for ambulation and bladder function have been externally validated on datasets not used to construct the original models [24, 25]. This article is the first study to successfully evaluate the external validity of the model for bowel function.

External validation of existing predictive models is seldom performed but is a crucial step before the predictive models can be applied to practice [11, 26]. Compared to constructing a new prediction model, external validation does not waste findings from previous works and results in less ‘model overload’ in the literature, which often leads to predictive models being ignored. Our work uses a rigorous methodological approach, elucidated in a 2014 critical review of external validation studies [26], to validate a recently built logistic regression model for bowel function after SCI. As recommended in the review, we use ROC curves, calibration curves, and decision curves to determine the discrimination, calibration, and clinical utility of the models, respectively.

The methodological rigor of our study and the successful external validation of the simplified model have important implications for clinical practice. Since the simplified model uses only the baseline total motor score as the predictor, accurately predicting a patient’s bowel function in the long-term is a relatively simple task that can be undertaken in a short clinical examination. This prediction will help patients and clinicians in being psychologically prepared well in advance and in initiating management strategies early on for neurogenic bowel dysfunction. This early initiation will lower rehabilitative costs and potentially improve outcomes in SCI patients. Further, early prediction of bowel outcomes will aid researchers designing clinical trials and prospective studies surrounding interventions for bowel dysfunction [10, 27].

This study is limited by its nature as a retrospective analysis of prospectively collected data. In particular, the use of data from a past cohort (when there were fewer available interventions for the sequelae of SCI) may limit the applicability of our external validation results to future cohorts. Additionally, there were some inconsistencies between the original EMSCI data used for model derivation and our North American SCI data. Our data primarily used version II of the SCIM questionnaire to quantify functional outcomes, while the EMSCI data used a combination of versions II and III. Further, the EMSCI study employed no intervention group as it was designed to evaluate the natural progression of SCI. However, two of the studies used for our analysis (NASCIS III and STASCIS) employed intervention groups (methylprednisolone and early surgery). Finally, our study is limited by the nature of the dataset, which had many missing patients since only a subset had complete SCIM outcomes. However, despite these limitations, the simplified model exhibited similarly good performance on both the original EMSCI cohort and our validation cohort, adding evidence that supports the model’s potential generalizability to clinical practice.

## Conclusions

We assessed the predictive performance of two logistic regression models predicting independence in bowel function 1 year after SCI. The simplified model, which used only baseline total motor score as the predictor, showed good discrimination and calibration on an external North American SCI dataset. Our study provides evidence supporting the use of this model to augment clinical practice, though continued external validation on additional prospectively collected data is needed to fully realize this goal.

## Supplementary information

Supplementary Table 1

## Data Availability

The dataset and computer code generated during and/or analyzed during the current study are available from the corresponding author on reasonable request.
