# Supplementary material for: Prediction of independence in bowel function after spinal cord injury: validation of a logistic regression model
Source: Spinal Cord. 2020 Sep 22;59(2):207–14. doi: 10.1038/s41393-020-00551-y (PMC7870806; doi:10.1038/s41393-020-00551-y)
Supplement: Supplementary file 1 — Supplementary Table 1 [file 41393_2020_551_MOESM1_ESM.docx]

**Supplementary Tables**

**Supplementary Table 1.** Relationship between probability predicted by simplified regression model and value of International Standards for Neurological Classification of Spinal Cord Injury (ISNCSCI) total motor score at baseline.

| **ISNCSCI Total Motor Score at Baseline** | **Predicted Probability** |
| --- | --- |
| 0 | 0.09530979 |
| 5 | 0.11847086 |
| 10 | 0.14634978 |
| 15 | 0.17945372 |
| 20 | 0.21813226 |
| 25 | 0.26248064 |
| 30 | 0.31224469 |
| 35 | 0.36675183 |
| 40 | 0.42489562 |
| 45 | 0.48519458 |
| 50 | 0.54592767 |
| 55 | 0.60532408 |
| 60 | 0.66176464 |
| 65 | 0.71394810 |
| 70 | 0.76098839 |
| 75 | 0.80243370 |
| 80 | 0.83822011 |
| 85 | 0.86858589 |
| 90 | 0.89397302 |
| 95 | 0.91493606 |
| 100 | 0.93206936 |
